# Osteoid osteoma of the acetabulum successfully treated with computed tomography-guided resection and ablation using a standard electrosurgical generator: a case report

**DOI:** 10.1186/s13256-016-1136-8

**Published:** 2016-12-03

**Authors:** Kazutaka Kikuta, Sota Oguro, Tatsuya Yamamoto, Tetsuya Sekita, Sayaka Yamaguchi, Michiro Susa, Kazumasa Nishimoto, Masanori Inoue, Seishi Nakatsuka, Aya Sasaki, Kaori Kameyama, Masaya Nakamura, Morio Matsumoto, Hideo Morioka

**Affiliations:** 1 Department of Orthopaedic Surgery, Keio University School of Medicine, 35 Shinanomachi, Shinjyuku-ku, 160-8582 Tokyo Japan; 2 Department of Radiology, Keio University School of Medicine, 35 Shinanomachi, Shinjyuku-ku, 160-8582 Tokyo Japan; 3 Department of Pathology, Keio University School of Medicine, 35 Shinanomachi, Shinjyuku-ku, 160-8582 Tokyo Japan

**Keywords:** Osteoid osteoma, CT guidance, Heat ablation, Acetabulum, Case report

## Abstract

**Background:**

Osteoid osteoma accounts for approximately 10% of all benign bone tumors. The most common sites of osteoid osteoma are the subcortical shaft and metaphyses of long bones, but any other skeletal bone site can be involved. The acetabulum is a rare site according to past reports. This site presents challenges to optimal management because it is anatomically difficult to approach, and because its rarity leads to limited experience with therapeutic procedures. Here, we report for the first time a rare case of osteoid osteoma in the acetabulum that was successfully treated via resection of the nidus and ablation using a standard electrosurgical generator under computed tomographic guidance.

**Case presentation:**

A 9-year-old Japanese girl presented at a clinic with left hip pain without apparent cause for 1 month. She was diagnosed as having coxitis simplex. However, her pain did not change for 1 year and she was admitted to another hospital where osteoid osteoma in her left acetabulum was suspected. She was then referred to our hospital approximately 1 year after first symptom presentation, where she presented with severe left hip pain and was completely unable to walk. Computed tomography examinations revealed a well-demarcated 5 mm mass with bone sclerosis in her left acetabulum. The mass was characterized by low intensity on T1 and high intensity on T2 magnetic resonance images. These findings were consistent with osteoid osteoma of left acetabulum. She underwent computed tomography-guided resection of nidus and ablation using a standard electrosurgical generator. A histological examination confirmed acetabular osteoid osteoma. Complete pain relief was achieved after the procedure and she experienced no complications. She could walk without any pain at the final follow-up 1 year post-treatment and no local recurrence was observed.

**Conclusions:**

We successfully treated acetabulum osteoid osteoma without any symptom recurrence by computed tomography-guided resection and ablation using a standard electrosurgical generator. In addition, we preserved our patient’s sciatic nerve and triradiate cartilage. Our report provides evidence that a computed tomography-guided procedure should be considered the treatment of choice for osteoid osteoma of the acetabulum because it is a less invasive alternative to *en bloc* resection.

## Background

Osteoid osteoma (OO) is a benign bone tumor characterized by a nidus with a maximum growth potential of 2 cm and surrounded by reactive sclerotic bone [1]. Pain is the presenting symptom. The pain is often nocturnal and usually responds to anti-inflammatory drugs such as aspirin. OO selectively develops in adolescents and young adults [2]. OO accounts for approximately 10% of all benign bone tumors. The most common sites are the subcortical shaft and metaphyses of long bones, such as the femur [3], but any other skeletal bone can be involved. The acetabulum is a rare site according to past reports, accounting for approximately 1% of OO lesions [4, 5]. The rarity of acetabular OO is a challenge to optimal management because of limited surgeon experience with therapeutic procedures. Another challenge to an approach is presented by its difficult anatomical location proximal to the sciatic nerve and triradiate cartilage. Over the past two decades, less invasive computed tomography (CT)-guided percutaneous surgical methods, including resection, drilling, and ablation, have superseded open *en bloc* resection for OO [6]. However, these procedures are technically challenging, with the acetabulum positioned near neurologic structures as well as juxta-articular and intra-articular localizations [7, 8]. In this report, we present a rare case of OO arising from the acetabulum that was successfully treated with CT-guided resection and ablation using a standard electrosurgical generator. In addition, we preserved the sciatic nerve, by making a small incision, and the triradiate cartilage using intraoperative images. No local recurrence or symptoms were observed at the latest follow-up 1 year post-treatment.

## Case presentation

**Fig. 1:**
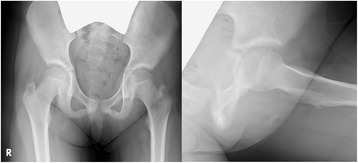
Patient radiographs on admission. Anteroposterior (*left*) and lateral (*right*) radiographs showing no obvious osseous abnormality

**Fig. 2:**
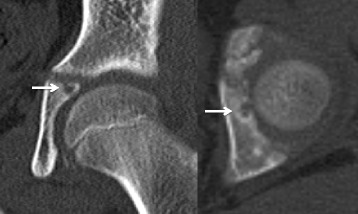
Patient computed tomography images on admission. Coronal view (*left*) and axial view (*right*) of computed tomography examinations showing a well-demarcated 5 mm mass surrounded by bone sclerosis (*arrows*) in the left acetabulum adjacent to the triradiate cartilage

**Fig. 3:**
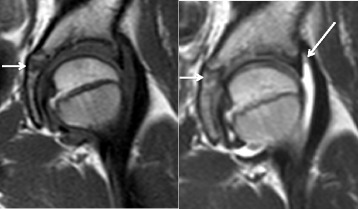
Patient magnetic resonance images. Coronal T1 (*left*) and T2 magnetic resonance images (*right*) showing that the mass was characterized by low intensity on T1 images and high intensity on T2 images (*arrows*). Magnetic resonance imaging also showed joint effusion (*long arrow*). Together with clinical findings and computed tomography images, the small lesion was diagnosed as osteoid osteoma of the acetabulum

**Fig. 4:**
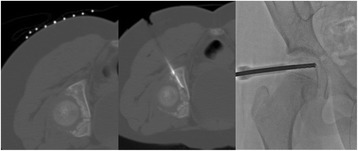
Intraoperative findings. In order to resect and ablate the nidus, a computed tomography-guided procedure was selected to minimize the invasiveness of surgery. First, the positional relationship between the nidus, triradiate cartilage, and sciatic nerve was identified using a computed tomography marker (*left*). After making a small incision to avoid the sciatic nerve, a guide pin was inserted toward the nidus. A 5.0 mm cannulated drill was inserted over the guide pin to remove the lesion and the specimen which resided in the cannulated drill was sent for histological study. Subsequently, heat ablation was performed using a standard electrosurgical generator to destroy any residual tumors (*middle*). To preserve the triradiate cartilage, the position of the electrode tip was confirmed during each step using intraoperative images (*right*)

**Fig. 5:**
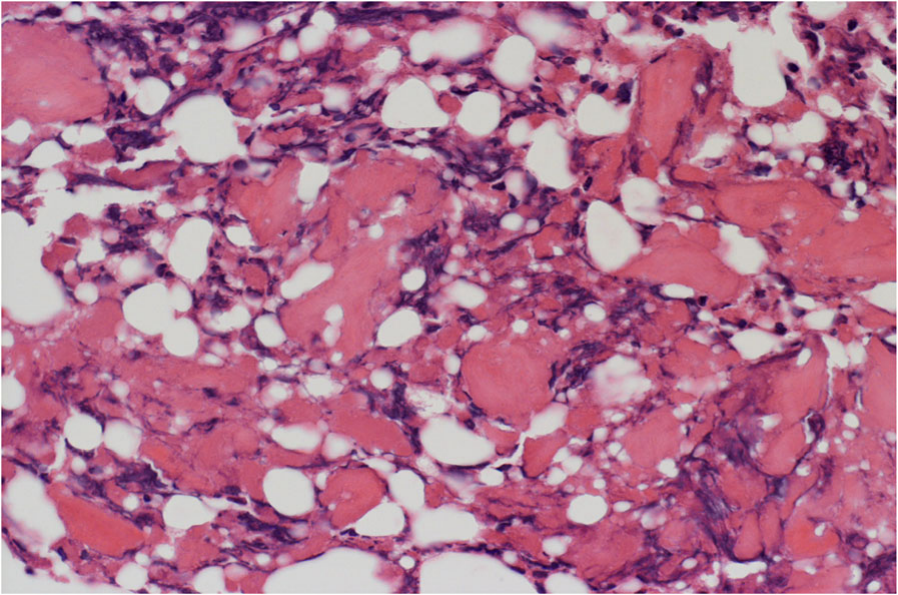
Histological findings. Histological findings showing random reticular osteoid formation within a fibrovascular stroma, consistent with osteoid osteoma

A healthy 9-year-old Japanese girl presented to a clinic with left hip joint pain without apparent cause for 1 month. She was diagnosed as having coxitis simplex. Although she had taken anti-inflammatory drugs, her pain did not change for 1 year. She was then sent to another hospital, where OO in her left acetabulum was suspected. As a result, she was referred to our hospital approximately 1 year after her first symptom presentation, where she presented with severe left hip pain and was completely unable to walk. Radiographs of her left hip showed no obvious osseous abnormality (Fig. 1). In contrast, CT examinations revealed a well-demarcated 5 mm mass with limited bone sclerosis in her left acetabulum (Fig. 2). The mass was characterized by low intensity on T1 and high intensity on T2 magnetic resonance imaging (MRI) images. MRI also revealed joint effusion (Fig. 3). Clinical and radiological findings were consistent with OO of the left acetabulum. She underwent CT-guided resection of the nidus and ablation using a standard electrosurgical generator at a power output of 15 W for 60-seconds duration (Fig. 4). This procedure was performed under total anesthesia and in the prone position. We identified the positional relationship between the nidus, triradiate cartilage, and sciatic nerve using a CT marker. After making a small 3-cm long incision while avoiding her sciatic nerve, a guide pin was inserted. A 5.0 mm cannulated drill was inserted over the guide pin to remove the nidus, which was then sent for histological examination. Next, ablation using a standard electrosurgical generator was performed to completely destroy any residual tumors. To preserve her triradiate cartilage, the position of the electrode tip was confirmed on intraoperative images during each step. The technical tips and pearls of CT-guided resection are summarized in Table 1. A histological examination confirmed the characteristic appearance of OO (Fig. 5). Complete pain relief was achieved beginning on the first postoperative day. Our patient could walk without any pain at the final follow-up 1 year post-treatment, and no local recurrence was observed.

**Table 1:** The technical tips and pearls of computed tomography-guided resection

Number	Step	Tips
1	Approach	Preoperative planning to assess the optimal approach to the nidus.
		It should be the shortest route to the bone surface and must avoid the neurovascular bundle.
2	Position	Patient must be positioned to allow easy access to the bone.
3	Incision	Blunt dissection should be performed to the bone to protect nerves and vessels. A small incision may be needed if an important structure is nearby.
4	Drilling	Drill hole perpendicular to the bone surface. Contralateral cortex should not be compromised to allow through ablation after resection of the nidus.
5	Fluoroscopy	Computed tomography usage should be minimized. Fluoroscopy is utilized to check the positioning of the guide pin and drill.
6	Diagnosis	The specimen inside the cannulated drill is important because it can be used to make a histological diagnosis.
7	Ablation	Standard electrosurgical generator is placed at the site of the lesion for 60 seconds under 15 W to ablate the possible remaining tumor cells.

## Discussion

The acetabulum is a rare OO site. According to prior reports [4, 9, 10], OO in the acetabulum accounts for only 0.67 to 4.85% of all OO sites. An OO diagnosis can be confirmed by a combination of images [5]. Plain radiographs can detect an oval, radiolucent central nidus surrounded by a dense, reactive sclerotic mass, specifically for cortical lesions. However, OO detection by plain radiography alone is difficult for intramedullary lesions or a location on the spine, pelvis, hands, or feet [11]. These locations are frequently associated with a delayed diagnosis, as was seen in our case. CT scans are the most accurate imaging technique for observing the nidus in complex anatomic sites such as the pelvis and spine.

OO is characterized by local pain that is more frequent and severe at night. Anti-inflammatory drugs, such as aspirin, are justified nonsurgical palliative treatments. For cases of severe pain with little to no response to pharmacologic treatment, surgical treatment is recommended to prevent developmental complications, such as growth disturbances [12].

Surgical treatment for acetabular OO is challenging because of limited surgeon experience with therapeutic procedures and the complex anatomical location proximal to the sciatic nerve and triradiate cartilage. Although numerous surgical approaches to acetabulum OO have been described, including open surgical hip arthroscopy and CT-guided approaches [5, 10], optimal management for acetabular OO has not been established.

In 1990, Voto *et al.* reported the successful treatment of OO by percutaneous CT-guided resection [13]. Over the past two decades, several CT-guided percutaneous treatments have been described, including drilling, radiofrequency ablation (RFA), ethanol injection, and a combination of these methods. These procedures are less invasive than open *en bloc* resection and have superseded open procedures [6].

Of these CT-guided procedures, CT-guided RFA has become the preferred method owing to its low morbidity rate, minimal postoperative complications, minimal tissue exposure, rapid recovery, and lack of restriction to weight-bearing activities. The healing rate is 76 to 100%, with a major complication rate of 0 to 5%. However, there are reports of articular cartilage damage in weight-bearing joints after CT-guided RFA: one patient experienced articular cartilage damage to the talus and another had damage to the acetabulum [5, 14]. In addition, because the system is expensive, percutaneous RFA is performed in only a few hospitals worldwide [15].

It was reported that CT-guided ablation for OO using a standard electrosurgical generator instead of an RFA system produced efficacy and safety results similar to those achieved with a RFA [15]. A standard electrosurgical generator is available in nearly all hospitals and can be more conveniently obtained than RFA systems.

The most severe complication following ablation of the acetabulum is a burn of the normal surrounding soft tissue, including the sciatic nerve and the triradiate cartilage. However, it was reported that the size of the ablated diameter using a standard electrosurgical generator at 15 W was only 5 mm, and that at 30 and 50 W it was only 9 mm. In addition, the duration of heat applied (30 to 120 seconds) did not make a difference to the size of the ablated lesion. Furthermore, an electrode of standard electrosurgical diameter did not protrude from the hole in the bone, and the temperature around the hole did not exceed 50 °C [15]. Therefore, in the present case, we performed ablation at a power output of 15 W for 60-seconds duration. In addition, we made a small incision to avoid our patient’s sciatic nerve, thus preventing its damage and burning during the procedure. Also, the position of the tip of the electrode in each step was confirmed by intraoperative images to prevent damage to her triradiate cartilage. Such images were also useful for decreasing her exposure dose. To the best of our knowledge, our patient is the first treated with CT-guided resection and ablation using a standard electrosurgical generator, a small incision, and intraoperative images. Complete pain relief was achieved beginning on the first postoperative day. She could walk without any pain at the final follow-up visit 1 year post-treatment, and no local recurrence was observed.

## Conclusions

In conclusion, we successfully treated acetabular OO without any symptom recurrence using CT-guided resection and ablation using a standard electrosurgical generator. In addition, we preserved the sciatic nerve and triradiate cartilage by making a small incision and using intraoperative images. A CT-guided procedure should be considered the treatment of choice for OO of the acetabulum because it is a less invasive alternative to *en bloc* resection.

## Abbreviations

CT: Computed tomography
MRI: Magnetic resonance imaging
OO: Osteoid osteoma
RFA: Radiofrequency ablation
